# The Fundamentals of Laparoscopic Surgery and LapVR evaluation metrics may not correlate with operative performance in a novice cohort

**DOI:** 10.3402/meo.v20.30024

**Published:** 2015-12-04

**Authors:** Sarah N. Steigerwald, Jason Park, Krista M. Hardy, Lawrence Gillman, Ashley S. Vergis

**Affiliations:** Department of Surgery, University of Manitoba, Winnipeg, Manitoba, Canada

**Keywords:** education, assessment, evaluation, selection, novice, simulation

## Abstract

**Background:**

Considerable resources have been invested in both low- and high-fidelity simulators in surgical training. The purpose of this study was to investigate if the Fundamentals of Laparoscopic Surgery (FLS, low-fidelity box trainer) and LapVR (high-fidelity virtual reality) training systems correlate with operative performance on the Global Operative Assessment of Laparoscopic Skills (GOALS) global rating scale using a porcine cholecystectomy model in a novice surgical group with minimal laparoscopic experience.

**Methods:**

Fourteen postgraduate year 1 surgical residents with minimal laparoscopic experience performed tasks from the FLS program and the LapVR simulator as well as a live porcine laparoscopic cholecystectomy. Performance was evaluated using standardized FLS metrics, automatic computer evaluations, and a validated global rating scale.

**Results:**

Overall, FLS score did not show an association with GOALS global rating scale score on the porcine cholecystectomy. None of the five LapVR task scores were significantly associated with GOALS score on the porcine cholecystectomy.

**Conclusions:**

Neither the low-fidelity box trainer or the high-fidelity virtual simulator demonstrated significant correlation with GOALS operative scores. These findings offer caution against the use of these modalities for brief assessments of novice surgical trainees, especially for predictive or selection purposes.

Surgical practice across disciplines continues to evolve and incorporate more technically and visually challenging procedures. There has subsequently been a great deal of attention towards means of improving technical skill education and even predicting an individual's future ability to perform these challenging procedures.

Some authors have gone as far as to suggest that surgical skill assessments can be used for selection purposes (1, 2). The idea of being able to use predictive tests to select those who may have a higher aptitude for surgery or specific disciplines is appealing for a number of reasons. First, it can help programs to select among prospective candidates, specifically those who are, or will be most adept at surgery. Second, it may guide candidates and help them decide if certain fields are appropriate for them. It is an unfortunate event to discover that inherent technical limitations preclude an individual from becoming a surgeon after investing years of pursuit and training.

Minimally invasive surgery (MIS) is a rapidly evolving and increasingly standard part of practice for gastrointestinal surgeons, and trainees preparing to enter practice must learn and master these techniques. MIS involves a distinct skill set compared to traditional open surgery, most significant of which includes working in three dimensions while working off two-dimensional video images, as well as adapting to the fulcrum effect, and less tactile feedback. Related to this distinct skill set, laparoscopy may require a more specific type of evaluation if that assessment is to be considered in any selection process (3, 4).

Considerable resources have gone into investigating the use of basic skills laboratories that incorporate surgical simulation as ways to teach and assess trainees in a safe environment outside of the operating room. Laboratory simulation–based skills training and assessment for laparoscopic surgical skills include lower fidelity physical box or video trainers and the higher fidelity virtual reality trainers. Both demonstrate significant correlations between operative performance and psychomotor performance in lab-based settings and can be used to assess operative laparoscopic skills (4–17). These physical box trainers are composed of frames supporting traditional laparoscopic video monitors, light sources and camera systems (18). Trainees use authentic laparoscopic instruments to perform tasks under videoscopic guidance (19). Virtual reality trainers use computer-generated images linked to a human–computer interface enabling users to perform simulated tasks (20). There is a spectrum of machines available, with the newer ones incorporating haptic feedback.

The video and virtual reality simulators provide the potential to assess the psychomotor and technical skills required to competently perform laparoscopic surgery outside of the operating room in a safe, controlled environment. Multiple previous studies have demonstrated that validated video trainer–based assessments and some virtual simulators are useful to discriminate between and predict interoperative performance across training levels. However, these studies often include comparisons of widely disparate cohorts, such as complete novices and expert surgeons, and it is difficult to know how well these methods evaluate performance within cohorts. There is limited investigation that evaluates physical box trainers and virtual reality systems for operative assessment purposes in novice groups with minimal laparoscopic experience. This is important as surgical training programs look to novel training, assessment, or even selection methods outside of the operating room. It is particularly problematic for novice groups, as resource and ethical considerations prevent unassisted operative skill observation using traditional human surgical models. This study aims to evaluate and compare the predictive performance of novice surgical trainees using the low-fidelity Fundamentals of Laparoscopic Surgery (FLS) box trainer and the high-fidelity LapVR simulator when assessed against the initial case of a live porcine model of laparoscopic cholecystectomy.

## Methods

### Participants

First-year urology and general surgery residents at the University of Manitoba were recruited to participate in this study. All subjects were novice operators with essentially no prior laparoscopic experience. Inclusion criteria included a lack of prior laparoscopic experience and availability to participate in the study.

Institutional ethics approval was obtained through The University of Manitoba Health Research Ethics Board for this investigation. Residents first completed a questionnaire outlining their prior laparoscopic experience and any practice on video trainers or virtual reality simulators.

### Simulation

Orientation to both the FLS and LapVR tasks with correct technique was provided. Residents performed one repetition each of four of the manual skills portion of the FLS tasks (peg transfer, pattern cutting, ligating loop, and intracorporeal knot tying). Residents also performed one repetition each of five of the level-one essential tasks on the LapVR virtual reality simulator that most closely approximated the FLS tasks (peg transfer, pattern cutting, clip application, needle driving, and knot tying).

### Porcine cholecystectomy

All participants performed a live porcine laparoscopic cholecystectomy. Subjects participated in a half-day course that consisted of lectures on the principles of laparoscopic surgery, a review of the relevant anatomy, and videos demonstrating surgical techniques prior to performing the cholecystectomy. The FLS tasks were done prior to the porcine cholecystectomy. Based on infrastructure availability, the LapVR simulation activities were then completed after. Activities were done as close in time as possible, and participants were surveyed with regard to any interim laparoscopic or simulator experience to account for potential confounders.

### Outcome measures

The main outcome measure assessed was the correlation or predictive validity between operative Global Assessment of Laparoscopic Skills (GOALS) score and the FLS and LapVR simulator scores.

GOALS is a valid and reliable assessment tool for evaluating laparoscopic skills (21) (Table 1). It consists of several skills domains, each graded on an anchored 5-point Likert scale. GOALS shows near total correlation to the Objective Structured Assessment of Technical Skills (OSATS) scores on recent investigation and is used in our skills laboratory to assess laparoscopic skill (22).

**Table 1:** Global Operative Assessment of Laparoscopic Skills (GOALS)

Performance	Rating				
	
characteristic	1	2	3	4	5
Depth perception	Constantly overshoots target, wide swings, slow to correct		Some overshooting or missing of target, but quick to correct		Accurately directs instruments in the correct plane to target
Bimanual dexterity	Uses only one hand, ignores non-dominant hand, poor coordination between hands		Uses both hands, but does not optimize interaction between hands		Expertly utilizes both hands in a complimentary manner to provide optimal exposure
Efficiency	Uncertain, inefficient efforts, many tentative movements, constantly changing focus or persisting without progress		Slow, but planned movements that are reasonably organized		Confident, efficient, and safe conduct, maintains focus on task until it is better performed via an alternative approach
Tissue handling	Rough movements, tears tissues, injures adjacent structures, poor grasper control, grasper frequently slips		Handles tissue reasonably well, minor trauma to adjacent tissue (i.e., occasional unnecessary bleeding or slipping of the grasper)		Handles tissue well, applies appropriate traction, negligible injury to adjacent structures
Autonomy	Unable to complete entire task, even with verbal guidance		Able to complete task safely with moderate guidance		Able to complete task independently without prompting

Modified from Vassiliou et al. (24). *Note*: 1=worst possible score, 5=best possible score.

FLS task performance was evaluated using standardized FLS metrics (4–6, 15). The LapVR generates automatic computer evaluation metrics. Parameters demonstrating validity evidence were then manually extracted (23) (see Table 3 in the Results section for specific parameters). A single academic minimally invasive surgeon who was blinded to the results of the FLS and LapVR scores assessed operative performance on the live porcine laparoscopic cholecystectomy using the GOALS rating scale. There were a total of four experienced laparoscopic surgeons involved with the rating process. An overview of how to use the rating tool was provided, and all were previously extensively trained in the use of the instrument. Additionally, two of the four raters participated in the primary and external validation of GOALS (21, 24). The surgeon did not interfere with the resident's conduct of the operative procedure. All forms of outcome measures have been previously validated.

### Data analysis

The analyses were carried out using Statistical Analysis System SAS 9.2 (SAS Institute, Cary, NC). All *p*-values were two tailed and a *p*-value <0.05 was considered statistically significant. Linear correlation (Pearson's coefficient) was calculated between GOALS and overall FLS scores. This could not be accomplished for LapVR scores as there is no summary metric for available. Thus, multivariable regression analysis was carried out to examine the association between outcome variable (GOALS score) and predictor variables (FLS overall score or LapVR data from each domain: peg transfer, cutting, clipping, needle driving, and knot tying). The *R* ^2^ from these multivariable regression analyses were used as a measure of how strongly FLS and LapVR evaluation metrics correlate with intraoperative performance. Continuous variables (e.g., GOALS score) were summarized in the descriptive analyses using the mean and standard deviations. Categorical variables (e.g., having comprehensive score) were summarized using the number (percent) in various categories.

## Results

All participants had minimal laparoscopic experience as the primary operator with an average of only 0.8 operations. Similarly, few had spent any significant amount of time practicing on a virtual simulator (average 0.4 h). The subjects did have more average time practicing on the video trainer (11.6 h); however, this number was elevated secondary to an outlier who had 150 h of practice. The rest of the residents had between 0 and 4 h of previous box trainer experience. The average days between activities were 29.9 with a range of 1–77. Interim laparoscopic experience was found to be more useful than days between activities, as residents were rotating on non-surgical rotations and not participating in any operative or simulation activities between sessions. Residents performed no interim laparoscopic procedures (average 0) and did not spend any time on the virtual reality simulator (average 0 h). They spent minimal time practicing on the video trainer with an average of only 1.1 h (Table 2).

**Table 2:** Participant characteristics

	Overall (*N*=14)
Age (mean)	29.2
Gender	
F, *n* (%)	8.0 (57.1)
M, *n* (%)	6.0 (42.9)
Specialty	
General surgery, *n* (%)	11.0 (78.6)
Urology, *n* (%)	3.0 (21.4)
Laparoscopic experience (procedures as primary operator) [average (range)]	0.8 (0–7)
Box trainer experience (h) [average (range)]	11.6 (0–150)
VR experience (h) [average (range)]	0.4 (0–3)
Days between laparoscopic cholecystectomy and LapVR simulation activities [average (range)]	29.9 (1–77)
Interim laparoscopic experience (as primary operator) between porcine cholecystectomy and LapVR activities [average (range)]	0 (0–0)
Interim box trainer experience (h) between porcine cholecystectomy and LapVR activities [average (range)]	1.1 (1–2.5)
Interim VR experience (h) between porcine cholecystectomy and LapVR activities [average (range)]	0 (0–0)

**Fig. 1:**
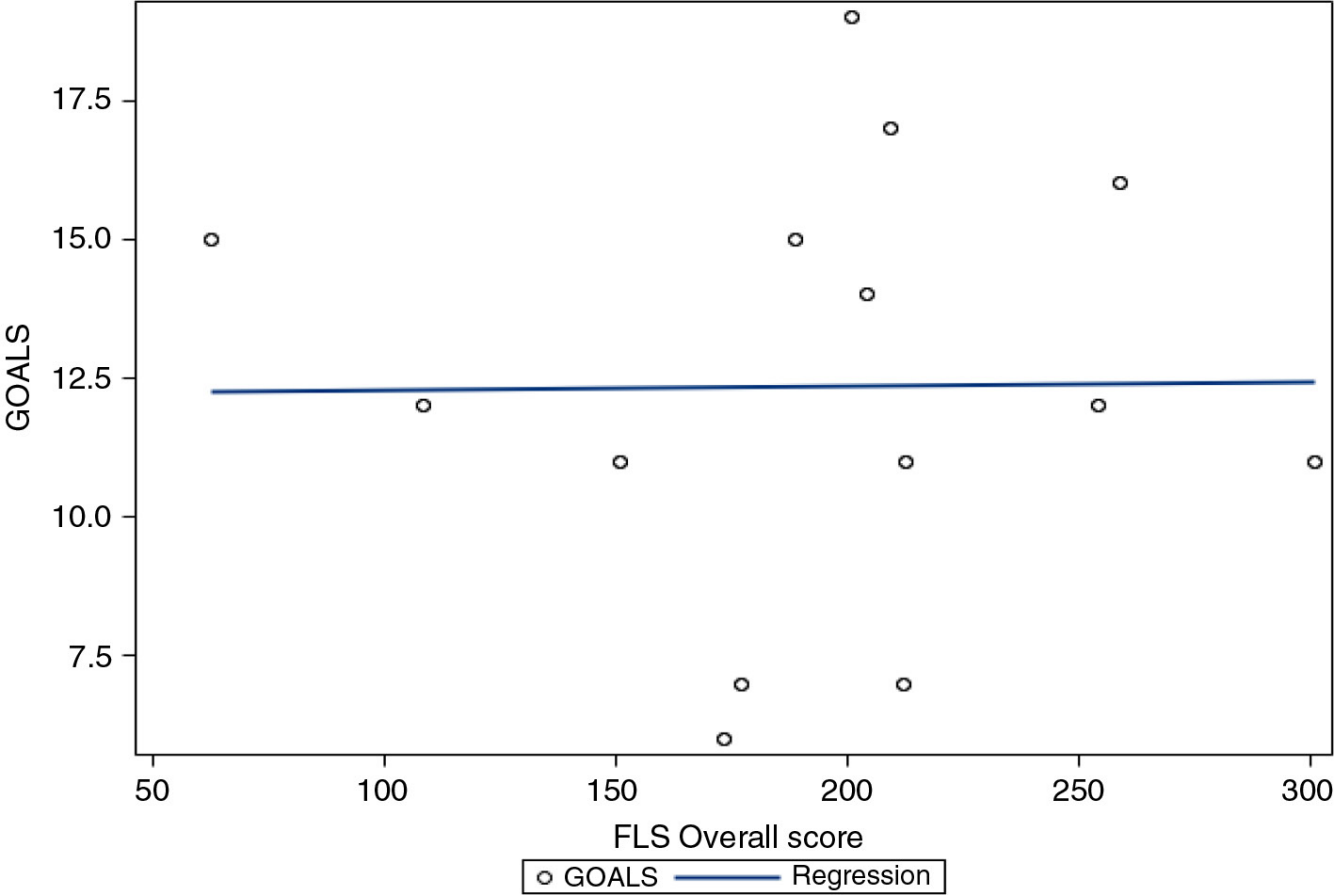
Relationship between FLS overall score and GOALS.

Figure 1 illustrates the relationship between overall FLS and GOALS scores (Table 3). There was no significant relationship demonstrated between overall FLS score and GOALS operative score. Similarly, for the LapVR, none of the five tasks demonstrated a significant relationship with GOALS operative score (Table 4).

**Table 3:** Descriptive statistics of outcome and predictor variables

	Overall score (*N*=14)
**GOALS**	21.50 (6.71)
**FLS**	
Peg transfer (time, s)	194.60 (28.40)
Pattern cutting (time, s)	22.93 (30.38)
Ligating loop (time, s)	83.89 (37.88)
Intracorporeal suturing (time, s)	232.03 (141.80)
**Overall score**	194.99 (60.55)
**LapVR**	
**Peg Transfer**	
Time (s)	145.20 (51.50)
Non-dominant time (s)	41.93 (18.00)
Non-dominant path length	3.14 (1.43)
Comprehensive score, yes/no (%)	7 (50%)
**Cutting**	
Time (s)	289.60 (156.60)
Unsuccessful cuts total	11.86 (8.40)
Non-dominant path length	2.58 (2.07)
Comprehensive score, yes/no (%)	6 (42.9%)
**Clipping**	
Time (s)	91.14 (24.47)
**Needle driving**	
Time (s)	426.90 (185.90)
Dominant path length	8.36 (3.59)
Non-dominant path length	5.79 (3.14)
**Knot tying**	
Time (s)	342.80 (171.20)
Dominant path length	7.91 (5.20)
Non-dominant path length	7.98 (5.43)

*Note:* Values reported are mean (standard deviation) unless specified.

**Table 4:** Multivariable regression analysis for GOALS

	*R*^2^	*p*
**FLS**		
Overall score	0.000	0.98
**LapVR**		
Peg transfer	0.48	0.17
Cutting	0.26	0.57
Clipping	0.05	0.43
Needle driving	0.49	0.07
Knot tying	0.43	0.15

## Discussion

The porcine model provides an opportunity for novice trainees to engage in a high fidelity *in vivo* operative model without attending surgeon interference as may be required for safety in a human model. This affords a novel uncontaminated assessment of trainee skill particularly in the novice.

Overall, FLS score did not correlate with initial GOALS global rating scale using a porcine laparoscopic cholecystectomy model. This is in contrast to previous literature that has demonstrated the predictive validity of FLS (5, 6, 9). This could potentially be the result of a sample size that was not large enough to show a true effect. However, it is more likely that the evaluation metrics employed may be unable to discriminate between subjects with little variation in their scores due to their nominal abilities. The studies referenced above include participants with a wide range of laparoscopic experience. Thus, the difference's realized between cohorts is not surprising. Additionally, in majority of prior studies, participants generally performed training sessions on the simulator prior to laparoscopic cholecystectomy performance. In the present study, our participants were completely novice operators with minimal prior formal simulator training and this was the first time many of them had been the primary surgeon for a laparoscopic case. As there was no attending surgeon take-over and the residents did not undergo training sessions on the simulators, the operative assessments were reflective of trainee skill and not influenced by staff surgeon involvement. The lack of correlation in this setting suggests that these simulation modalities are not useful forms of assessment in groups who are expected to have minimal skill sets.

The LapVR tasks had to be compared to overall operative score individually as currently there is no validated overall summary score for the tasks as in FLS. Additionally, the individual LapVR tasks could not be compared to the individual FLS tasks as each LapVR task had anywhere from one to four scoring parameters contributing to the task evaluation. None of five LapVR tasks correlated with GOALS operative score. Predictive validity has been demonstrated for virtual reality simulators; however, the literature primarily looks at the MIST VR virtual simulator and the LapSim virtual reality simulator (7, 8, 10–13). For this study, correlation was not shown for the LapVR and GOALS operative score. Again, the lack of demonstration of correlation may be related to the novice level of the residents group as operators.

Only a limited number of studies have focused on how simulation-based assessments can be applied to more purely novice groups of trainees. A Brazilian study demonstrated predictive validity between a group of ‘novice’ operator's laparoscopic colectomy and LAP Mentor (Simbionix, Cleveland, OH) simulation skills (25). However, in this study, all subjects had previously independently performed laparoscopic procedures such as cholecystectomy, appendectomy, and fundoplication. Our investigation is unique in that the novice cohort essentially had negligible prior laparoscopic experience. This questions whether a completely untrained group of operators should be assessed using FLS or VR simulation metrics. These results have implications on early assessment of surgical trainees within training programs. They also caution the use of these forms of assessment in candidate selection, as some authors have suggested (1, 2).

## Limitations

This study has numerous strengths, including a relatively homogeneous cohort of novice surgery residents, the assessment of skill using previously validated outcome measures, and the assessment of operative performance on a live porcine model. This last point is important because it allowed us to assess each participant as they performed an entire *in vivo* cholecystectomy dissection (not just parts of the procedure), but it allowed us to do so in a controlled and safe environment without ‘attending take-over’ of any parts of the case for patient safety reasons. Several limitations, however, require discussion. First, this study has a limited number of participants (*N*=14). A follow-up study with a larger sample size would help delineate if any differences exist between the two forms of simulation. Second, there is no overall comprehensive LapVR score as in FLS. Therefore, individual LapVR tasks must be compared to overall FLS score. The development of an overall LapVR score would allow a more direct comparison between the two forms of simulation (26). Third, there was some variability in the time between laparoscopic cholecystectomy and simulator tasks. Given the complicated nature of surgical residents’ schedules, opportunity sampling was used. Thus, setting up a specific time frame between the activities was not possible. However, there were no interim laparoscopic procedures performed or virtual simulator experience by any of the participants. As well, interim video trainer experience was limited with subject's performing an average of 1.1 h. Additionally, due to the nature of the lab set up and resident availability, the LapVR tasks had to be completed at a later date. We attempted to control for this as the FLS and laparoscopic cholecystectomy were a one-time intervention and there was no interim training before the LapVR tasks; however, we do recognize this is a potential limitation. Lastly, due to time constraints each participant was only evaluated by one rater. While there is the potential for this to decrease the reliability of the results, the internal consistency and inter-rater reliability of GOALS have been well demonstrated in the past (24).

## Conclusions

Neither the low-fidelity box trainer nor the high-fidelity virtual simulator demonstrated significant correlation with GOALS scores in this investigation. These findings offer caution against the use of these modalities for brief assessments of novice surgical trainees, especially for predictive or selection purposes.
